# Psychometric properties of the Big Three Perfectionism Scale‐Short Form (BTPS‐SF) among Iranian University students

**DOI:** 10.1002/brb3.3227

**Published:** 2023-08-17

**Authors:** Zahra Neshat, Azam Farah Bijari, Gholamreza Dehshiri

**Affiliations:** ^1^ Department of Psychology, Faculty of Education and Psychology Alzahra University Tehran Iran

**Keywords:** Big Three Perfectionism Scale‐Short Form, Psychometric Properties, University Students

## Abstract

**Introduction:**

Perfectionism is a common personality trait, particularly among university students. As awareness of this trait increases, research seeks to update tools for measuring it. The psychometric properties of the three‐dimensional perfectionism scale‐short form (BTPS‐SF) have not been studied in the Iranian population. Therefore, this study aims to investigate the psychometric properties of this 16‐item scale among Iranian university students.

**Methods:**

The statistical population for this research included all students in Tehran. From this population, 528 students were selected, and data analysis was performed using SPSS 26 and AMOS 24 software.

**Results:**

The findings showed that the BTPS‐SF has acceptable validity and reliability. Furthermore, a positive and significant correlation was found between perfectionism and interpersonal sensitivity, whereas a negative and significant correlation was found between perfectionism and self‐compassion.

**Conclusion:**

Given the desirable psychometric properties of the Persian version of the BTPS‐SF, its use can be recommended to psychology experts in research and clinical evaluation situations.

## 1 INTRODUCTION

For university students, success in academic life is considered important because it affects other aspects of life (Çapan, 2010). Academic success is influenced by various factors, including social (such as parental engagement and support, peer relationships, adjustment, and socioeconomic status), cognitive (such as language proficiency and academic self‐efficacy), personal (such as motivation, time management), educational (such as lack of information), and emotional (emotional well‐being) (Thomas & Maree, 2021). In the academic success of students, not only the mentioned factors are effective but personality factors are also effective (Zhou, 2015). In research, the positive effect of personality traits, such as conscientiousness (Hazrati‐Viari et al., 2012), openness (Zhou, 2015), extraversion, and agreeableness (Lievens et al., 2002) on academic success, has been proposed. Another personality trait that affects academic performance and learning is perfectionism (Chasetareh et al., 2023; Madigan, 2019). The concept of perfectionism has been defined in different ways over time. In general, perfectionism is a personality trait in which a person sets tough standards for himself and tries hard to complete his actions flawlessly (Flett & Hewitt, 2002).

According to Beck's (1976) perfectionism cognitive model, many mental disorders occur due to certain errors in perceptual information processing, commonly known as cognitive distortions. Conceptually, perfectionism is consistent with cognitive models of psychopathology and represents a maladaptive cognitive style (Pacht, 1984). Perfectionists have an all‐or‐nothing thinking pattern and tend to see events in black or white. Accordingly, all‐or‐nothing thinking causes them to fear mistakes and overreact to them (Burns, 1980).

Understanding the difference among the real self (characteristics that a person actually has), the ideal self (characteristics that a person want to have), and the imperative self (characteristics that a person is responsible for) is abundantly experienced in perfectionistic people that can cause all kinds of emotional distress (Higgins, 1987). Slade and Owens (1998) also suggested regarding the self‐concept of perfectionists that positive perfectionists use perfectionism to get closer to their ideal selves. These people believe that if they reach their standards, it will be rewarding and there will be no negative consequences if they do not. However, people with negative perfectionist characteristics set goals with the idea that even if these goals are achieved, failure is imminent, if they are not achieved, negative consequences follow, and they do not enjoy achieving goals.

In addition to the concept of perfectionism, the construct of perfectionism has also undergone changes in the past decades in such a way that at first they had a one‐dimensional view of this structure and considered it negative (Stoeber, 2018). In unidimensional perfectionism, perfectionists are defined as those who have standards beyond reach, compulsively and relentlessly strive to achieve impossible goals and measure their worth entirely in terms of productivity and success (Burns, 1980). Unidimensional perfectionism measures a person's perfectionistic beliefs using a 10‐item scale and shows perfectionism on a continuum. Researchers have now rejected this approach and have since adopted multidimensional models. Multidimensional models consider perfectionism as a complex construct with different components or factors (Lauber, 2013).

Hamachek (1978) proposed an early multidimensional approach to perfectionism. He considered two aspects of perfectionism: The first dimension is positive or normal perfectionism, which means accepting one's limitations while trying to become better. The second dimension is neurotic perfectionism, which has a pathological aspect in that it causes a person to worry about making mistakes, fear judgment, and dissatisfaction with personal performance (Slade & Owens, 1998).

Since the 1990s, perfectionism has been considered a multidimensional construct that can have both positive and negative characteristics (Stoeber, 2018). Hewitt and Flett (1991) and presented a model of perfectionism that is defined by three dimensions. Self‐oriented perfectionism is formulated based on preexisting concepts about perfectionism, that is, according to the views of Burns and Beck (1978), Hamachek (1978), Pacht (1984), and Higgins et al. (1986). Self‐oriented perfectionism means setting exact standards for oneself and evaluating oneself based on these standards, and a motivational component has been added to this dimension by Hewitt and Felt (1991), which means continuous effort to achieve success and avoid failure. Other‐oriented perfectionism, which is the same as self‐oriented perfectionism, but is directed toward others and society, means considering high standards for important people in life and a critical view of their performance, and finally, socially prescribed perfectionism, which was first introduced by Hewitt and Felt (1991) systematically investigated as meeting the strict standards of others that the individual considers these standards important. Each of the three dimensions of perfectionism is related to different structures in clinical and student groups.

One of the variables related to perfectionism is self‐compassion (Shahidi Delshad et al., 2023; Tan, 2023) and another variable is interpersonal sensitivity (Mohammadian et al., 2018). Regarding perfectionism dimensions, the self‐related dimension in perfectionists can be related to self‐compassion because self‐critical individuals may view self‐compassion as exaggeration, selfishness, or even weakness (Gilbert, 2009). Since perfectionists believe that they must be fully competent, sufficient, and intelligent in all aspects, they do not accept themselves as beings with human limitations and errors (Ellis, 1958). This makes people feel worthless by overgeneralizing their mistakes (Hewitt & Flett, 1991). The other‐related dimension of perfectionism can be related to interpersonal sensitivity because perfectionism is associated with a need for social approval and a fear of negative evaluation (Flett & Hewitt, 2004). A strong need for perfection makes a person fearful of being criticized and tries to hide his or her flaws from others and becomes overly sensitive to criticism (Hu & Bentler, 1999).

Apart from the correlation of perfectionism with self‐compassion and interpersonal sensitivity, this personality trait is related to other types of variables related to mental health in students (Visvalingam et al., 2022) and many researchers consider the effect of perfectionism on educational outcomes to be important (Zhang et al., 2007). Perfectionism in students causes harm, such as procrastination (Çapan, 2010), depression (Dorevitch et al., 2020), decreased psychological well‐being (Ko et al., 2020), academic anxiety (Dobos et al., 2021), decreased life satisfaction (Sharifi et al., 2018), academic burnout (Garratt‐Reed et al., 2018), increases shame (Dorevitch et al., 2020). Therefore, considering the importance of this structure and its consequences, a basic need with psychometric study aims to assess perfectionism in order to protect students from the mentioned dangers by early detection.

So far, various tools have been developed to measure perfectionism, and these tools measure different aspects of perfectionism. Smith and colleagues (Smith et al., 2016) created a 45‐item scale named Big Three Perfectionism Scale (BTPS) to measure the global criteria of perfectionism, which was most adapted from the perspective of Hewitt and Felt (1991). This questionnaire includes three higher‐order global factors called rigid perfectionism, self‐critical perfectionism, and narcissistic perfectionism that are evaluated by 10 lower‐order perfectionism facets called self‐oriented perfectionism, self‐worth contingencies, concern over mistakes, doubts about actions, self‐criticism, socially prescribed perfectionism, other‐oriented perfectionism, hypercriticism, grandiosity, entitlement (Smith et al., 2016).

Rigid perfectionism means pursuing one's flawless performance and includes self‐oriented perfectionism and self‐worth contingencies, the first of which means pursuing and being perfect, and the second of which means feeling self‐esteem based on having high standards. Self‐critical perfectionism includes concern over mistakes (negative and excessive reaction to failures), doubts about actions (uncertainty of one's own performance), self‐criticism (severe criticism of oneself when the desired performance is not achieved), and socially prescribed perfectionism (others want the individual to be perfect) (Smith et al., 2016). Finally, narcissistic perfectionism means demanding perfection from others through other‐oriented perfectionism (setting high standards for others), hypercriticism (excessive criticism of others’ performance), grandiosity (believing that one is perfect and superior to others), and entitlement (the belief that a person is perfect and should be treated in a special way) (Dunkley et al., 2003). BTPS is the only questionnaire that can measure people who believe that they are perfect and superior to others, in other words, they are narcissistic perfectionists. Narcissistic perfectionists see no difference between their ideal self and their actual self, which makes them feel entitled and ultimately provokes criticism from others (Smith et al., 2016).

In their research, Feher et al. (2019) created a short English version of BTPS, called BTPS‐Short Form (SF). This scale is a 16‐question scale that, like its long version, measures the three factors of rigid perfectionism, self‐criticism, and narcissism. The results of the research indicate the validity and reliability of this questionnaire among Canadian students in such a way that by considering three factors, the number of fit indices showed a favorable evaluation because to determine the factors, one‐factor and two‐factor models: rigid perfectionism of a factor and narcissistic perfectionism had a poor fit, but the three‐factor model had the most appropriate fit, and the factor loadings of the three‐factor model were estimated from 0.49 to 0.92. It has good test–retest reliability as the correlation level from .71 to .79 is significant at the .001 level (rigid perfectionism, *r* = .79, *p* < .001, self‐critical perfectionism, *r* = .75, *p* < .001 and narcissistic perfectionism, *r* = .71, *p* < .001) Cronbach's alpha also varies from .78 to .90. Moreover, the perfectionism factors showed the expected divergent validity and convergent validity with the scales measuring personality traits, trait emotional intelligence, resiliency, life satisfaction, positive and negative affect, depression, stress, and anxiety. Thus, rigid perfectionism and self‐criticism had a positive relationship with neuroticism, rigid perfectionism, self‐criticism, and narcissism had a negative relationship with agreeableness and conscientiousness, and rigid perfectionism had a negative relationship with conscientiousness. Moreover, emotional intelligence had a negative relationship with rigid perfectionism and self‐criticism; resiliency had a negative relationship with three factors of perfectionism; there has been a negative relationship between rigid perfectionism and self‐criticism with life satisfaction; three factors of perfectionism had a positive relationship with negative affect, and self‐critical perfectionism had a negative relationship with positive affect, and finally, three factors of perfectionism had a positive and significant correlation with depression, anxiety, and stress (Feher et al., 2019).

In their research, Kaçar‐Başaran et al. (2022) standardized the short form and long form of this scale among 427 Turkish adults and concluded that this scale has acceptable reliability and validity. To check the reliability, the test–retest method was used, and the correlation of the factor of rigid perfectionism was 0.80, self‐critical perfectionism was 0.87, and narcissistic perfectionism was estimated at 0.79. The correlation of the perfectionism scale with the Frost Multidimensional Perfectionism Scale has been estimated as positive and high (Kaçar‐Başaran et al., 2022). Di Fabio et al. (2018) in their research examined the BTPS short form with 18 questions using the Mokken analysis method in Italian workers and concluded that the three‐factor structure has the best fit and Cronbach's alpha of all three factors estimated from .86 to .92, which indicates the appropriate reliability of the scale. Due to the wider use of the three‐factor structure with 16 questions by Fehr et al. (2019), the psychometric properties of this questionnaire were investigated in this research.

Considering the need for a new scale that measures a new dimension of perfectionism called narcissistic perfectionism, the purpose of this research is to standardize this scale among Iranian students. Also, due to the necessity of aligning measurement scales with the progress of research in various fields of psychology, it is important to examine the psychometric characteristics of the present questionnaire in Iranian society; Therefore, in this study, the 16‐item short version of BTPS was translated by using the Acquadro method (Acquadro et al., 2003) and the results of the concordance between the original version and the translation were examined that there was a necessary match.

## 2 METHODS

### 2.1 Participants

Participants were 528 university students from Tehran with an average age of 23.77 and a standard deviation of 3.24, whose ages ranged between 18 and 31. Overall, 49.4% (*n* = 261) of the students were female, and 50.6% (*n* = 267) were male. Overall, 24.1% of the students were in the humanities subfield (*n* = 127), and 75.9% of the students were in the mathematics subfield (*n* = 401). Moreover, 5.5% (*n* = 29) were at the postgraduate level, 40.7% (*n* = 215) were at the undergraduate level, 49.8% (*n* = 263) were at the master level, and 4% (*n* = 21) were studying for a doctorate.

### 2.2 Instruments

#### 2.2.1 The Big Three Perfectionism Scale (BTPS‐SF)

The short form of the perfectionism scale (Feher et al., 2019) is a 16‐item scale graded on a 5‐point Likert scale. The scale ranges from completely disagree 1 to completely agree 5. It includes three scales of rigid perfectionism, self‐critical perfectionism, and narcissistic perfectionism. Cronbach's alpha in the two studied samples ranged between .78 and .90 for perfectionism scales. Items 1–4 measure rigid perfectionism, 5–10 measure self‐critical perfectionism, and 11–16 measure narcissistic perfectionism (Feher et al., 2019).

To translate the English version of the BTPS‐SF scale into Persian using the method of Acquadro et al. (2003), the English scale was first translated into Persian by three people who are fluent in both the source and destination languages. Among the three, the best translations were selected by an expert. In the second stage, the Farsi version was translated into English by a translator, and no differences were observed between the translated version and the original version. Finally, the Persian BTPS‐SF scale was administered to five students. Ambiguities were resolved, and the final version was approved based on the lack of ambiguities for the students.

#### 2.2.2 Self‐Compassion Scale‐Short Form (SCS‐SF)

The short form of the Self‐Compassion Scale (Raes et al., 2011) is a scale consisting of 12 items, which includes three bipolar subscales. The first subscale is self‐kindness (items 2 and 6) versus self‐judgment (items 11 and 12). The second subscale is common humanity (items 5 and 10) versus isolation (items 4 and 8). The third subscale is mindfulness (items 3 and 7) versus overidentification (Sh et al., 2015). The scale is scored on a five‐point Likert scale, with a score of one indicating almost never and a score of five indicating almost always. The scores of items 1, 4, 8, 9, 11, and 12 are scored in reverse (Sh et al., 2015). Cronbach's alpha coefficient for the factors, that is, self‐kindness versus self‐judgment, common humanity versus isolation, and mindfulness versus overidentification, were reported as .79, .71, and .68, respectively, and Cronbach's alpha of the whole scale was estimated at .86 (Khanjani et al., 2016). In the present study, Cronbach's alpha of this scale is estimated at .80.

#### 2.2.3 Interpersonal Sensitivity Measure (IPSM)

The Interpersonal Sensitivity Measure is a questionnaire consisting of 36 questions and 5 subscales. The subscales are interpersonal awareness (items 2, 4, 10, 23, 28, 30, and 36), need for approval (items 6, 8, 11, 13, 16, 18, 20, and 34), separation anxiety (items 1, 12, 15, 17, 19, 25, 26, and 29), timidity (items 3, 7, 9, 14, 21, 22, 32, and 33), and fragile inner‐self (items 5, 24, 27, 31, and 35) (Boyce & Parker, 1989). The measure is graded on a four‐point Likert scale, with one indicating completely disagree and four indicating completely agree. The measure does not have reverse scoring, and the range of scores is between 36 and 144. A person's score that is close to 144 indicates high interpersonal sensitivity, and the closer it is to 36, the lower the interpersonal sensitivity. Cronbach's alpha of the whole measure is .86, and for the subscales, Cronbach's alpha for interpersonal awareness is .76, need for approval is .51, separation anxiety is .58, timidity is .58, and fragile inner‐self is .70 (Mohammadian et al., 2017). In the present study, Cronbach's alpha value of this measure is estimated at .87.

### 2.3 Procedure

Participating students answered the questions voluntarily. They were studying in four post‐diploma, bachelor's, master's, and doctorate fields related to technical, mathematical, and human sciences. A total of 528 questionnaires were provided to students online. Before starting, the participants expressed their consent to answer and were informed about the purpose and method of conducting the study. They were assured that the private and personal information of the candidates would be protected, and they could withdraw from the study if they wished. Furthermore, participating in the research did not have any financial burden for the participants, and finally, the results of the study were interpreted for them if they wished.

## 3 RESULTS

Primary data screening was done using AMOS 24 software. Since it was necessary to answer each of the items to submit the online questionnaires, there were no missing data. Outliers were evaluated using Mahalanobis distance in AMOS software. By dividing the maximum value of the Mahalanobis distance (45.99) by the number of questions, which is 16, a value of 2.87 was obtained. According to Tabachnick and Fidell (2014), a value of less than four is acceptable, and there were no outliers in the dataset. The univariate normality of the data was determined using skewness and kurtosis values. The analysis results showed that the lowest value of skewness was −1.18, and the highest value was 0.83. The lowest value of elongation was −1.12, and the highest value was 0.75. Therefore, considering that these values are less than ±2, the hypothesis of single‐variable normality is confirmed (Kline, 2015).

Cronbach's alpha was used to measure the reliability of the questionnaire. Cronbach's alpha coefficient was calculated for a rigid factor of .74, self‐criticism of .81, and narcissism of .75. Based on the opinion of Lance et al. (2006), Cronbach above .70 is acceptable. Since the Cronbach alpha value of the questionnaire is higher than .70, the reliability of the considered scale is acceptable.

The method of factor analysis, convergent, and divergent validity was used to check the validity of the perfectionism questionnaire. Bartlett's sphericity test and Kaiser–Meyer–Olkin's index were used to determine sample adequacy and reducibility. Bartlett's sphericity test showed that the chi statistic is 2676.48, and the significance level is .001. The Kaiser–Meyer–Olkin index showed a value of .85, which is greater than .70. So, according to the two mentioned results, the sample size is adequate, and there is a possibility to reduce the data. Therefore, factor analysis can be used.

**FIGURE 1:**
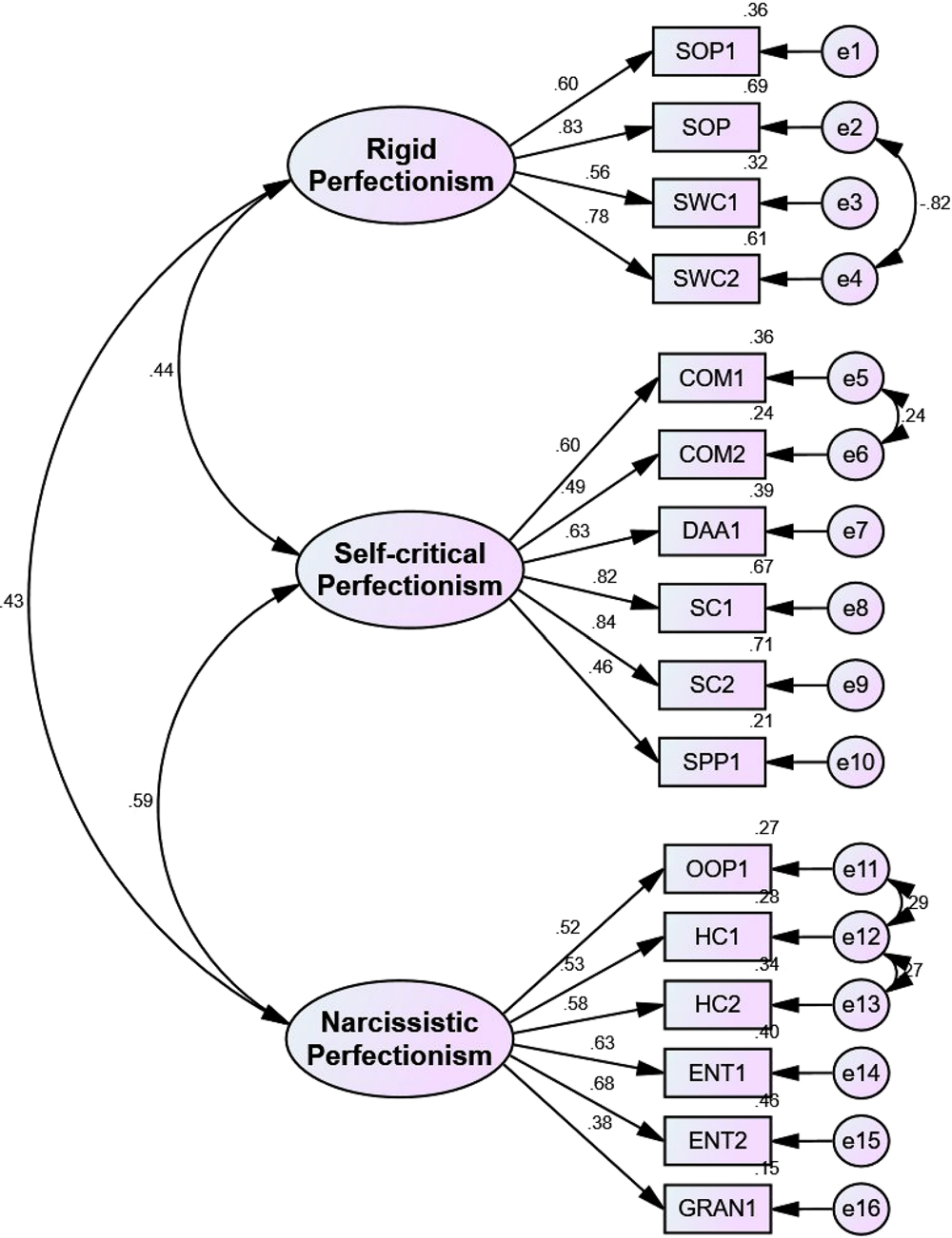
Confirmatory factor analysis of three‐factor Big Three Perfectionism Scale‐Short Form (BTPS‐SF). COM, concern over mistakes; DAA, doubts about actions; ENT, entitlement; GRAN, grandiosity; HC, hypercriticism; OOP, other‐oriented perfectionism; SC, self‐criticism; SOP, self‐oriented perfectionism; SPP, socially prescribed perfectionism; SWC, self‐worth contingencies.

To verify the validity of the structure, the factor loadings of all indicators fall between 0.38 and 0.84 (Figure 1). The results of CFA show that the factor loadings of all indicators are greater than 0.3 and less than 1, and none of them are negatively estimated. A factor loading of more than 0.30 indicates a moderate correlation between the item and the factor (Tavakol & Wetzel, 2020), implying a correlation between the items and the factor.

In the initial model without correcting the amount [*CMIN*/*df* = 4.51, *p* < .001, GFI = .90, IFI = .86, CFI = .86, RMSEA = .08, SRMR = .07], it was estimated that after correcting correlated errors, the indices the mentioned fit was improved, and the values of these indicators are mentioned below.

Table 1 shows the average and standard deviation of all indicators. The value of fit indices [*CMIN/df* = 3.36, *p* < .001, GFI = .92, IFI = .91, CFI = .91, RMSEA = .06, SRMR = .06] has been estimated and according to the criterion [*CMIN/df* < 5, GFI > .90, IFI > .90, CFI > .90, RMSEA < .1, SRMR < .09] is within the acceptable range (Hu & Bentler, 1998), so the fit of the measurement model was confirmed.

**TABLE 1:** Means and standard deviations of the items of the three‐factor Big Three Perfectionism Scale‐Short Form (BTPS‐SF).

No	Items	Mean	Std. deviation
1	I have a strong need to be perfect	3.35	1.28
2	It is important to me to be perfect in everything I attempt	3.90	1.14
3	Striving to be as perfect as possible makes me feel worthwhile	3.90	1.05
4	My opinion of myself is tied to being perfect	3.24	1.13
5	The idea of making a mistake frightens me	3.34	1.16
6	When I notice that I have made a mistake, I feel ashamed	3.75	1.02
7	I have doubts about everything I do	3.12	1.22
8	I judge myself harshly when I don't do something perfectly	3.27	1.16
9	I feel disappointed with myself, when I don't do something perfectly	2.96	1.17
10	People are disappointed in me whenever I don't do something perfectly	2.46	.98
11	I expect those close to me to be perfect	2.32	1.17
12	I am highly critical of other people's imperfections	2.55	1.17
13	I feel dissatisfied with other people, even when I know they are trying their best	2.15	1.13
14	It bothers me when people don't notice how perfect I am	2.52	1.20
15	I deserve to always have things go my way	3.17	1.25
16	I know that I am perfect	2.16	1.06

Abbreviations: AVE, average variance extracted; CR, composite reliability.

The construct reliability of the three factors of rigid perfectionism, self‐critical perfectionism, and narcissistic perfectionism is examined using the AVE. The AVE value of three factors is estimated as .49, .43, and .31, respectively. Although these values are lower than .50, the value of CR is estimated to be .79, .81, and .72, respectively, as per (Fornell & Larcker, 1981), and these values are higher than .6. As a result, the construct reliability (CR) is acceptable (Table 2).

**TABLE 2:** Average variance extracted (AVE), composite reliability (CR), and Cronbach's alpha for three‐factors of Big Three Perfectionism Scale‐Short Form (BTPS‐SF).

Variables	AVE	CR	Cronbach's alpha coefficient
Rigid perfectionism	.49	.79	.74
Self‐critical perfectionism	.43	.81	.81
Narcissistic perfectionism	.31	.72	.75

The interpersonal sensitivity measure was used for convergent validity, and the Self‐Compassion Scale was used for divergent validity. Table 3 shows the correlation of the three‐factor perfectionism scale with interpersonal sensitivity and with the Self‐Compassion Scale. Therefore, as expected, the correlation of the three factors of perfectionism with interpersonal sensitivity is positive and significant and with self‐compassion is negative and significant, as a result, the convergent and divergent validity of the perfectionism scale is confirmed.

**TABLE 3:** Correlations between variables.

Variables	1	2	3	4	5
1. Rigid perfectionism	1				
2. Self‐critical perfectionism	.37^*^	1			
3. Narcissistic perfectionism	.31^*^	.42^*^	1		
4. Interpersonal sensitivity	.23^*^	.64^*^	.41^*^	1	
5. Self‐compassion	−.61^*^	−.60^*^	−.37^*^	−.55^*^	1

* All correlation is significant at *p* *≤* .01 level.

## 4 DISCUSSION AND CONCLUSION

Personality psychology seeks to investigate individual differences in thoughts, feelings, and behaviors that remain constant in different times and places (Roberts & Yoon, 2022). Personality traits play a role in explaining students’ academic performance (Mammadov, 2022). One of the personality traits that can have a negative effect on the academic and social performance of students is perfectionism (Awad et al., 2022). Both the concept of perfectionism and the structure of perfectionism have gone through changes. The concept of perfectionism from a cognitive point of view is a network of cognitions that includes expectations and interpretations related to events and a person's evaluation of themself and others, along with unrealistic standards, insisting on these standards and equating one's worth with performance according to these standards (Burns, 1980). The main difference between striving for healthy success and striving for negative success lies in the interpretation of performance. When positive perfectionists achieve success, they are satisfied with their performance. However, those who display more neurotic perfectionism are never satisfied with their performance, even if they have successfully achieved a goal (Slade & Owens, 1998).

The structure of perfectionism has been investigated in a one‐dimensional form (Burns, 1980) and in a multidimensional form (Lauber, 2013) in subsequent research. In one‐dimensional perfectionism, people set unreasonable and unrealizable standards and goals for themselves (Burns, 1980). In the multidimensional view, positive perfectionists are individuals who are motivated to do their best without fear of failure (Hamachek, 1978), but neurotic perfectionists engage in an unhealthy form of striving for success that is accompanied by fear of failure and efforts to avoid performance errors are defined (Hall, 2006). The scale of positive and negative perfectionism of Terry‐Short et al. (1995) measures perfectionism based on this point of view, and the multidimensional questionnaire of Hewitt and Flett (1991) also measures perfectionism by considering three dimensions: self‐oriented perfectionism, other‐oriented perfectionism, and socially prescribed perfectionism. Another multidimensional assessment scale of perfectionism is the BTPS, which measures perfectionism as three dimensions of rigid, self‐criticism, and narcissism (Smith et al., 2016), and the BTPS‐SF is shortened based on this multidimensional perspective (Feher et al., 2019). Therefore, the aim of the present study was to investigate the psychometrics of the short form of the BTPS in Iranian students.

Since perfectionists expect high performance from themselves or others in various situations (Hollender, 1965), this personality trait plays a major role in a wide range of psychopathologies (Frost et al., 1990). One of the harms resulting from this personality trait is the reduction of self‐compassion (Ferrari et al., 2018) because they do not have unconditional acceptance of themselves (King & Pennebaker, 1998) and when failures, with harsh judgments toward others. The self and worrying about evaluations cannot be kind to oneself (Pereira et al., 2022). In the present study, to examine the divergent validity of the correlation between perfectionism and self‐compassion, rigid perfectionism had the most negative and significant correlation with self‐compassion because people with rigid perfectionism have a strong insistence on perfection and their self‐worth is conditional on achieving success and they punish themselves when they do not reach their high standards (Smith et al., 2016). Therefore, this maladaptive cognition causes them to judge and blame themselves by perceiving the difference between the goals they want and what they have achieved (Fletcher et al., 2019).

Another harm associated with perfectionism is an increase in interpersonal sensitivity (Mohammadian et al., 2018). Therefore, to examine the convergent validity, the correlation between perfectionism and interpersonal sensitivity was examined, and self‐critical perfectionism had the most positive and significant correlation with interpersonal sensitivity because perfectionists with self‐criticism emphasize the negative aspects of events and consider ordinary events as threatening (Hewitt & Flett, 1993) and perceive their own effectiveness in these situations as low and expect criticism from others in dealing with makes them adopt an avoidance approach (Carver, 1998). In the review of convergent and divergent validity, the results obtained in this research, similar to the research of Feher et al. (2019), three factors of perfectionism showed different correlations with other scales.

In addition to convergent and divergent validity, the results showed that the Persian version (BTPS‐SF) is suitable for a significant assessment of perfectionism among Iranian students. Exploratory factor analysis showed that the sample size is sufficient and it is possible to reduce the factors to three factors. Feher et al. (2019) and Kaçar‐Başaran et al. (2022) concluded in their research that the three‐factor model had the most suitable fit, so the three‐factor model was used in the present study. In the confirmatory factor analysis, the fit of the measurement model was confirmed after the correction because the fit indices were within the acceptable range and the factor loadings were estimated to be higher than .30.

Cronbach's alpha coefficient of rigid perfectionism, self‐critical perfectionism, and narcissistic perfectionism were .74, .81, and .75, respectively. This value is in line with the research results of Feher et al. (2019) and Kaçar‐Başaran et al. (2022). The value of composite reliability (CR) was higher than .60, which confirmed the reliability of the structure, although the value of the average variance extracted index was estimated below .5, because this index is a strict index the value of the CR index was used to determine the reliability of the structure.

In general, considering the need to coordinate psychological assessment measures with the advances in their concepts, this study also investigated the psychological characteristics of a new perfectionism scale in Iranian students. This scale can be used by researchers and therapists active in the field of psychology due to its ease of use, short number of questions, easy scoring, and validity in Iranian culture and society.

This research, like other research, has limitations, such as the fact that people tend to present themselves in a positive way, so this scale may not measure the real level of perfectionism in people's real life. Also, since the sample of this research is the students of Tehran city, caution should be observed in generalizing its results to other strata of society. For future research, it is suggested to examine this scale with other personality questionnaires and to use other reliability methods such as test–retest reliability or parallel forms. Also, check this scale in other samples of society, such as people who are middle‐aged or old, so that comparisons between different periods of life can be made possible.

## AUTHOR CONTRIBUTIONS

**Zahra Neshat**: Conceptualization; Methodology; Project administration; Validation; Formal analysis; Investigation; Resources; Data curation; Writing—original draft; Writing—review and editing and Visualization. **Azam Farah Bijari**: conceptualization; Supervision; Investigation; Writing—original draft; Writing—review and editing and visualization. **Gholamreza Dehshiri**: Methodology; Validation and Formal analysis.

## CONFLICT OF INTEREST STATEMENT

The authors declare no conflicts of interest.

## FUNDING INFORMATION

No funding was received for conducting this study.

### PEER REVIEW

The peer review history for this article is available at https://publons.com/publon/10.1002/brb3.3227.

## Data Availability

Data will be available by the corresponding author upon reasonable request.
